# Sphenopalatine ganglion stimulation for the treatment of trigeminal neuropathic pain

**DOI:** 10.3389/fneur.2025.1535102

**Published:** 2025-05-14

**Authors:** Paweł Sokal, Sara Kierońska-Siwak, Marcin Rusinek, Magdalena Jabłońska, Antoni Nehring, Oskar Puk, Damian Palus, Renata Jabłońska

**Affiliations:** ^1^Department of Neurosurgery, Functional and Stereotactic Neurosurgery, Faculty of Health Sciences Collegium Medicum in Bydgoszcz, Nicolaus Copernicus University in Toruń, Toruń, Poland; ^2^Doctoral School Collegium Medicum, Nicolaus Copernicus University, Bydgoszcz, Poland; ^3^Department of Neurological and Neurosurgical Nursing, Faculty of Health Science, Collegium Medicum in Bydgoszcz, Nicolaus Copernicus University in Toruń, Toruń, Poland

**Keywords:** sphenopalatine ganglion stimulation, trigeminal neuropathic pain, neuromodulation, chronic pain, trigeminal neuralgia

## Abstract

**Introduction:**

Unlike idiopathic trigeminal neuralgia, which can be treated with conventional neurosurgical methods such as microvascular decompression, radiofrequency rhizotomy of the Gasser ganglion, or stereotactic radiosurgery, trigeminal neuropathic pain (TNP) presents a major challenge for neurosurgeons. Injury to the trigeminal system resulting in chronic refractory pain can be treated with neuromodulation methods, such as peripheral nerve stimulation, motor cortex stimulation, or deep brain stimulation. Sphenopalatine ganglion (SPG) stimulation has been successfully applied in patients with cluster headaches and migraine. This study aimed to evaluate the response of patients with TNP to permanent percutaneous SPG stimulation.

**Methods:**

We studied six patients treated with SPG stimulation for TNP. All patients had previously been treated with RF rhizotomy, microvascular decompression, or stereoradiosurgery without a satisfactory long-term therapeutic effect and had recurrent, mostly constant TNP. An electrode lead was implanted in the pterygopalatine fossa of all patients to stimulate the SPG under guidance of neuronavigation with an implantable pulse generator inserted after a two-week trial period.

**Results:**

Preoperatively, the mean visual analog scale score was 9. Two weeks after the trial stimulation, it decreased to 3.6 in six patients. In four patients, the score further decreased to 3.0 after 6 months and 2.25 after 12 months, accompanied by an improvement in health status, as measured by the 36-Item Short Form Health Survey questionnaire. In two patients, the electrodes were externalized through eroded skin after 3 months, and stimulators were removed.

**Discussion:**

The preliminary results of this pilot study are encouraging. Pain relief after the trial stimulation was found to be notable. The treatment procedure was safe, and the stimulation effect was durable. SPG stimulation is an attractive alternative to other neuromodulation methods.

## 1 Introduction

Trigeminal neuropathic pain (TNP) is a chronic pain condition resulting from injury or dysfunction of the trigeminal nerve. When an injury to the trigeminal system leads to numbness and constant pain and is characterized by intense burning, shooting, or stabbing sensations, it is referred to as TNP (1). The etiopathogenesis may be associated with previous procedures, such as rhizotomy, gangliolysis, or nucleotomy (2). In cases of medication-resistant neuralgia, invasive approaches, such as nerve blocks, radiofrequency ablation, or neuromodulation devices, may be considered. Unlike idiopathic trigeminal neuralgia, which is often effectively managed with conventional neurosurgical procedures, such as microvascular decompression (MVD), radiofrequency (RF) rhizotomy of the Gasser ganglion, or stereotactic radiosurgery, TNP poses a significant challenge for neurosurgeons (1, 3–5). In cases of trigeminal deafferentation pain, in which the second and third neurons of the trigeminal-cortical pathway are injured, motor cortex or deep brain stimulation can provide meaningful pain relief (6, 7). The sphenopalatine ganglion (SPG) plays a central role in the pathophysiology of the autonomic etiology of headaches, such as trigeminal autonomic cephalgia (8). Located in the pterygopalatine fossa, the SPG receives sensory input from the second branch of the trigeminal nerve (V2) (9). SPG stimulation has emerged as a promising treatment for autonomic headaches, including migraines and cluster headaches, using on-demand neuromodulation devices (8, 10–12).

This study aimed to evaluate the response to permanent conventional percutaneous SPG stimulation in patients with TNP. It also assessed the effectiveness of this neuromodulation technique in patients with chronic neuropathic pain that is refractory to conventional treatments.

## 2 Materials and methods

This retrospective study included six patients with chronic TNP (five females and one male; average age, 58 years; range, 34–73 years). These patients experienced chronic pain refractory to conventional treatments for an average duration of 6 years (range 2–20 years), affecting the V2, V3, or V1 branches, typically following surgery, MVD, or zoster infection. Patients were previously treated with pharmacological methods, trigeminal nerve blocks, RF ablation of the Gasser ganglion, or botulinum toxin injections. Despite these methods, satisfactory pain control was not achieved in these patients. Six patients qualified for SPG stimulation, five of whom had TNP, and one had also features of anesthesia dolorosa. The detailed patient characteristics are presented in Table 1. All participants provided informed consent for treatment. The study adhered to the principles of the Declaration of Helsinki and was approved by the local Bioethics Committee. Patients were evaluated before surgery, 2 weeks after trial stimulation, and 6 and 12 months after stimulator implantation. Pain was assessed using the Visual Analog Scale (VAS), and health status was evaluated using the 36-Item Short Form Health Survey (SF-36) for the objective measure of the health-related quality of life, which describes physical functioning, role physical, bodily pain, general health, vitality, social functioning, role emotional, and mental health in 36 items (13).

**Table 1:** Patient demographics and medical history details.

Patient	JAW	EB	KW	MM	SC	WP
Age	48	65	61	34	68	73
Gender	F	F	F	F	M	F
Type of pain	TNP, (anesthesia dolorosa)	TNP	TNP	TNP	TNP	TNP
Side of the pain	Left	Left	Left	Left	Left	Right
Location of the pain	V2, V3	V2, V3	V2	V1, V2	V2, V3	V1, V2
Duration of symptoms	7 years	11 years	5 years	2 years	4 years	20 years
History of pain	Neurovascular conflict	No history of trauma, infections, surgeries	MVD	2× FESS	Zoster virus infection	Dental treatment
Complications	—	—	—	Electrode externalization after 3 months	—	infection and removal
Past medical treatment	1× MVD, 2× RFG	2× botox injection, 1× RFG, 1× lidocaine block	MVD, 2× RFG, 2× cryotherapy	2× lidocaine block, 1× RFG	2× lidocaine block, 1× RFG	1× RFG

F, female; M, men; FESS, functional endoscopic sinus surgery; MVD, microvascular decompression; TN, trigeminal neuralgia; TNP, trigeminal neuropathic pain; RFG, radiofrequency rhizotomy of Gasser ganglion.

Inclusion criteria were as follows:

1. Patients with persistent trigeminal neuropathic pain.
2. Patients who were not candidates for microvascular decompression or radiofrequency (RF) Gasserian rhizotomy.
3. Patients who provided informed consent for a two-stage stimulator implantation.
4. Patients with chronic neuropathic pain lasting at least 6 months, with a pain severity score greater than 5 on the Visual Analog Scale (VAS), significantly impacting daily functioning and quality of life.
5. Patients whose pain was inadequately managed with analgesic medications.

Exclusion criteria were as follows:

1. Patients with typical trigeminal neuralgia.
2. Patients whose neuropathic pain was successfully managed with analgesics.
3. Patients receiving anticoagulant therapy or those with coagulation disorders.

Due to the nature of the procedure, patients with significant contraindications to surgery—such as severe circulatory insufficiency, poorly controlled diabetes mellitus, coagulopathies, or those receiving anticoagulant or antiplatelet therapy—were excluded from the study. Additionally, elderly patients with significant anesthetic risk were not considered eligible.

### 2.1 Procedure

**Figure 1:**
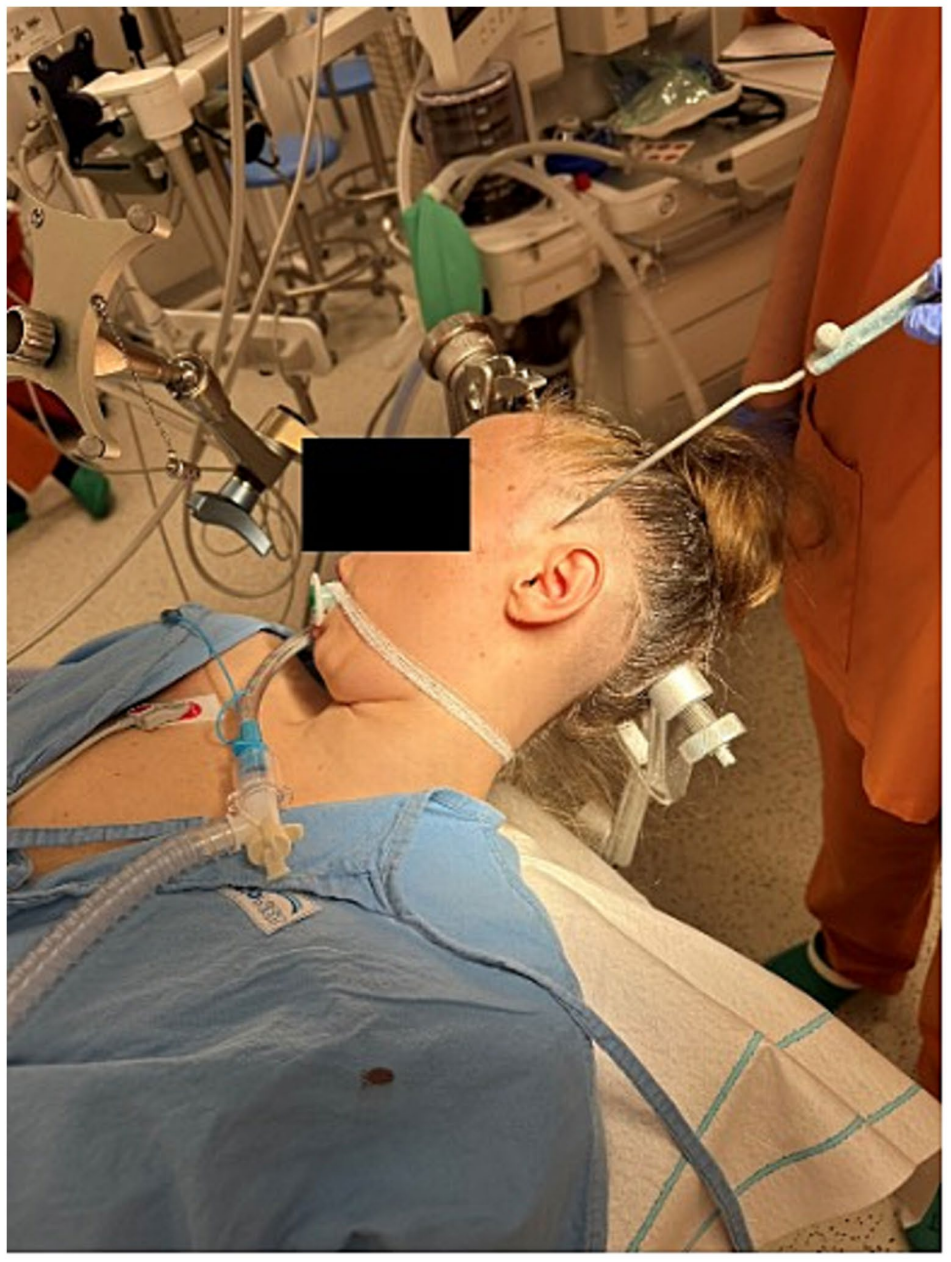
Electrode lead implantation procedure performed under general anesthesia with neuronavigation guidance (Stealth, Medtronic, United States).

**Figure 2:**
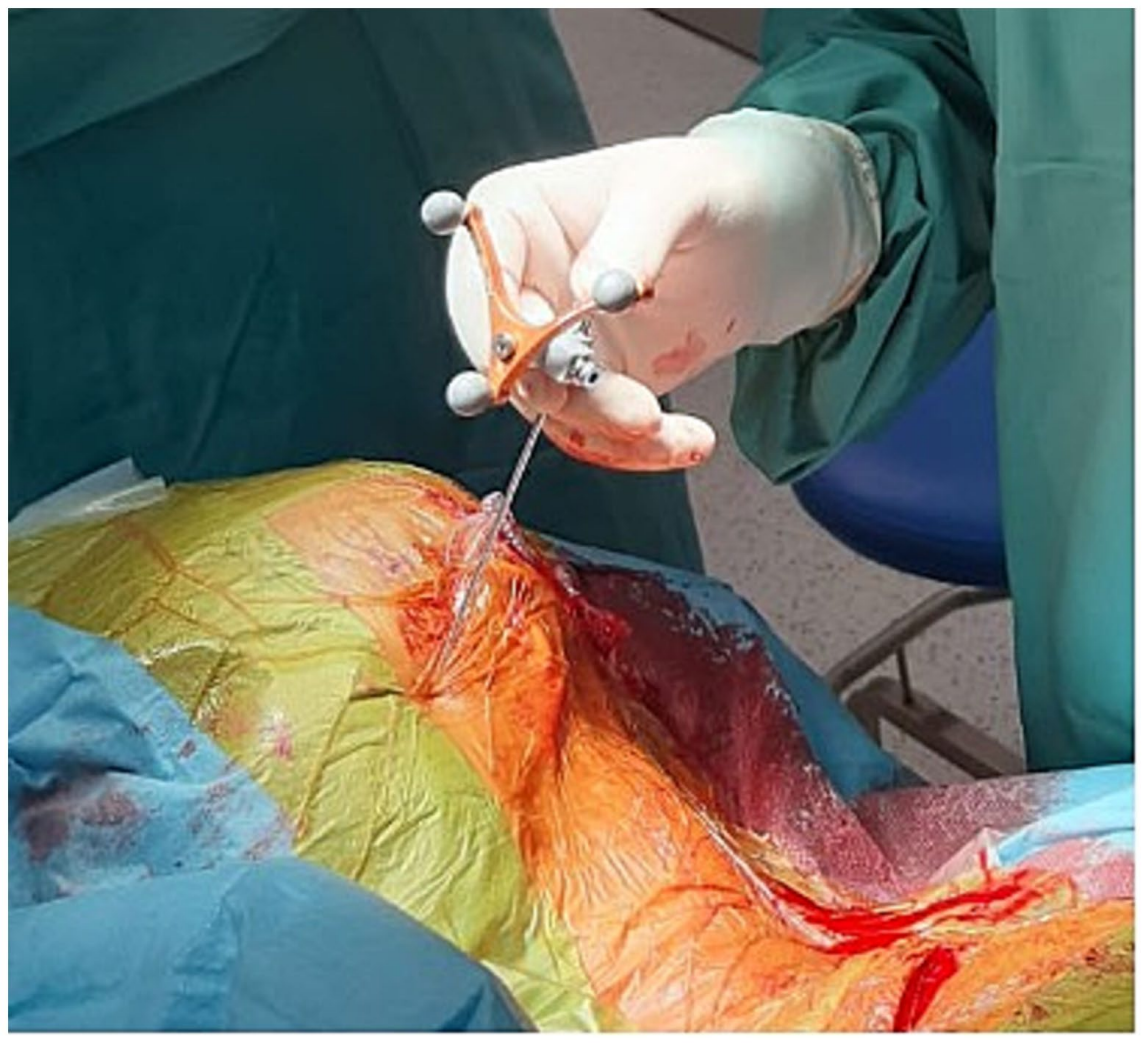
A navigable needle with a stylet guiding the trajectory for lead insertion.

**Figure 3:**
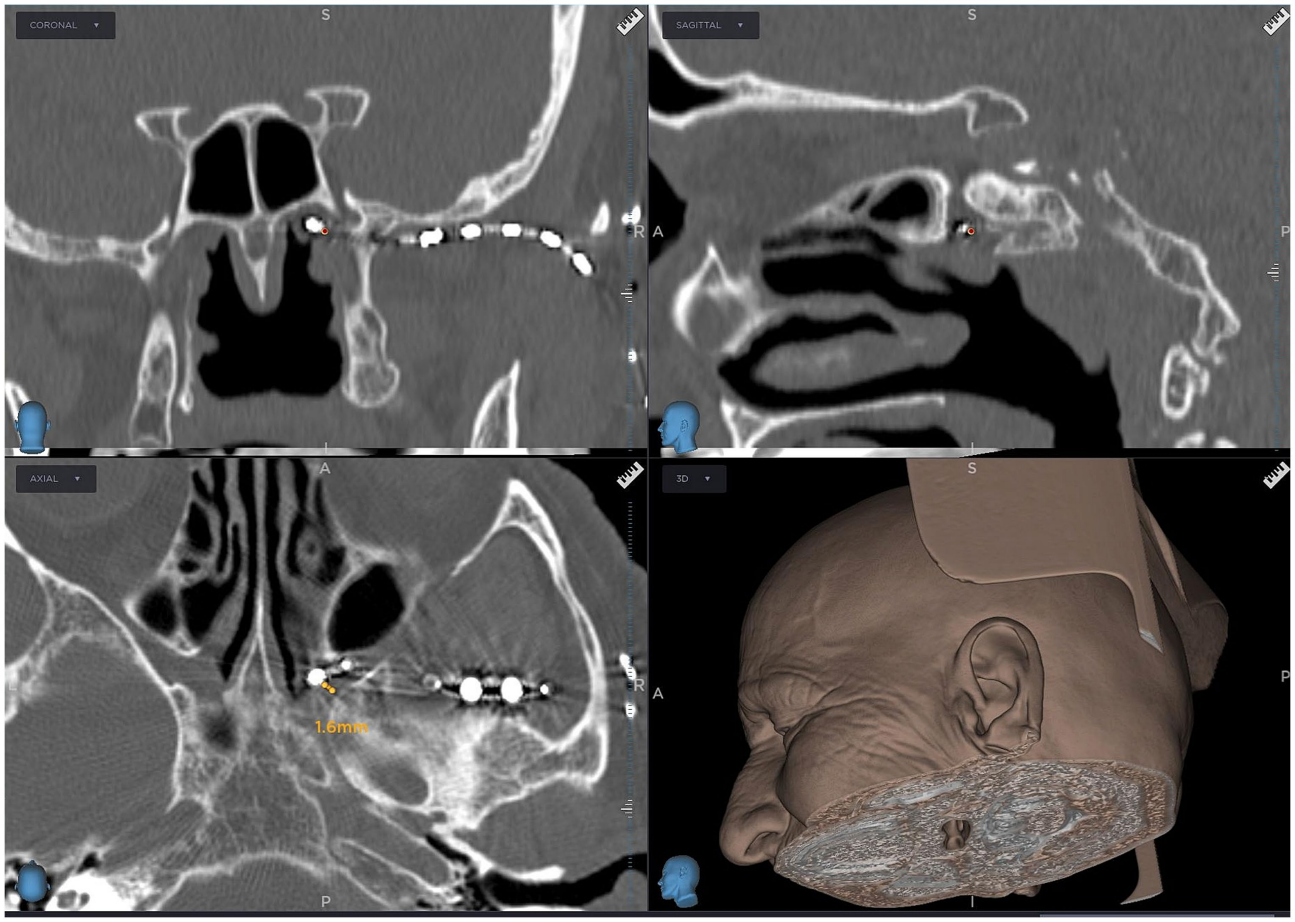
Computed tomography scan showing an 8-contact electrode lead implanted in the pterygopalatine fossa near the sphenopalatine ganglion, guided by neuronavigation (Stealth, Medtronic, United States).

Surgeries were performed under general anesthesia. A 14 G navigable needle with a stylet was introduced through the pterygopalatine fossa in the proximity of SPG and positioned behind the maxillary sinus and anterior to the pterygoid process of the sphenoid bone (Figures 1, 2). An 8-contact percutaneous electrode lead (Boston Scientific Inc., Marlborough, MA, United States or Abbot Inc., Austin, TX, United States) was inserted into the pterygopalatine fossa on the ipsilateral side of the pain under neuronavigation (Stealth, Medtronic, United States) based on preoperative computed tomography scans (Figure 3) and predicted localization of SPG (14). The lead was tunneled subcutaneously into the mastoid area, connected to an extension in the retromastoid area, and then connected to an external stimulator. After trial stimulation, the extension was connected to an implantable pulse generator (Boston Scientific Inc., United States or Abbot Inc., United States) and placed in the subcutaneous pocket in the subclavicular area. The first two contacts were activated with the parameters of tonic stimulation (Table 2). Control computed tomography scan of the head was performed postoperatively to determine lead localization.

**Table 2:** Tonic stimulation was administered to all patients, with the corresponding stimulation parameters detailed below.

Patient	Parameters of tonic stimulation
JAW	30 Hz, 500 μs, 2 mA
EB	30 Hz; 500 μs, 1.4 mA
KW	30 Hz; 500 μs; 2.3 mA
MM	40 Hz; 800 μs; 2.5 mA
SC	40 Hz, 500 μs; 2.2 mA
WP	40 Hz 400 μs; 0.40 mA

## 3 Results

Four of the six patients continued to receive SPG stimulation for the entire 12-month follow-up period and demonstrated satisfactory outcomes. Two patients (MM) and (WP), however, had only a 3-month follow-up due to device-related complications, including erosion and infection, which necessitated early discontinuation of the therapy.

All patients were closely monitored, and the use of standard pharmacologic treatments such as gabapentin, pregabalin, or tramadol was documented. However, the primary focus of the study was to evaluate the efficacy of SPG stimulation. The majority of patients discontinued these medications due to adverse effects, leading them to opt for neuromodulation therapy. In some cases, patients continued to use immediate rescue analgesics, such as tramadol, during subsequent SPG stimulation.

All patients underwent a minimum two-week trial, during which a pain reduction of at least 50% was achieved. The mean preoperative Visual Analog Scale (VAS) score was 9. After 2 weeks of stimulation, the mean VAS score decreased to 3.6, to 3.0 in four patients at 6 months, and further to 2.25 at 12 months. In this study, no minimum sample size was established.

**Figure 4:**
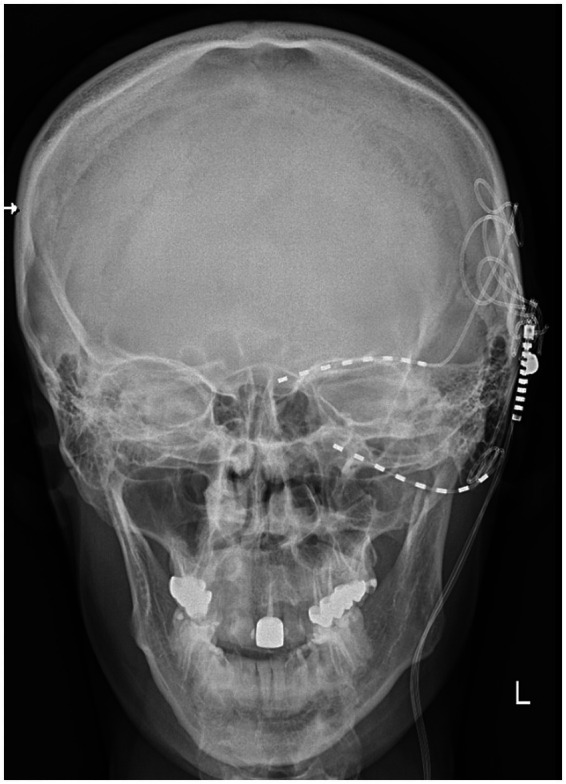
X-ray image showing the additional electrode lead positioned above the eyebrow for supraorbital nerve stimulation and the electrode lead in the pterygopalatine fossa in the patient (MM).

For patient (JAW), the VAS score decreased from 8 preoperatively to 3 at both the 6- and 12-month follow-ups. The patient (EB) reported a reduction in VAS score from 9 to 3 at 6 months and to 2 at 12 months following electrode implantation. In the patient (KW), pain decreased to 3 after 6 months and to 2 after 12 months. The patient (MM) initially presented with neuropathic pain affecting the V1 and V2 branches of the trigeminal nerve. Following the implantation of the electrode and battery, pain in the V2 branch was reduced by more than 50%, while pain in the V1 branch remained unchanged. Four weeks later, an additional electrode was implanted in the supraorbital region (Figure 4). However, the electrode ceased functioning after 3 months and was subsequently removed along with the battery. For the patient (MM), the VAS score assessed at 3 months postoperatively showed a reduction in pain, from a preoperative score of 10 to 3. Unfortunately, further follow-up was not possible due to electrode extrusion. The patient (SC) experienced a reduction in VAS score from 9 to 4 after 2 weeks of stimulation, with further improvement to a score of 2 at the 12-month follow-up. The patient (WP) underwent trial stimulation with a meaningful pain reduction from 10 to 4 on the VAS, followed by permanent implantation of the pulse generator.

Health status, as measured by the SF-36, improved from a mean score of 50 preoperatively to 70 after 2 weeks, and further to 80 during the follow-up period in four patients. The maximum SF-36 score before surgery was 60 points in two patients describing their pain on the VAS scale as 9 and 10, respectively. The minimum SF-36 score was 30 in a patient with pain defined as 10 points. The mean SF-36 score before surgery in the study group of patients was 50, while the median was 50.

After surgery, there was also an increase in the score in the SF-36 questionnaire, in which the minimum value was 60, the maximum 80. Both the mean and median for SF-60 after 2 weeks of the trial period were 70. After 6 months of stimulation, the SF-36 score increased to 80 points in all patients studied. Compared to the two-week trial period, the improvement in the SF-36 was 20 points in one patient, 10 points in two other patients, and did not change and amounted to 80 points in another. No further changes in the SF-36 score were observed, the value of which remained at a constant level of 80 points in all patients.

A comparison of pain intensity and health status is presented in Table 3.

**Table 3:** Results of SPG stimulation on pain intensity, as measured by the Visual Analog Scale (VAS), and overall health status, assessed using the SF-36, were evaluated after 2 weeks of trial stimulation at 6 and 12 months post-implantation.

Patient	Preoperative VAS	Preoperative SF-36	VAS 2 weeks	SF-36 2 weeks	VAS 6 months	SF-36 6 months	VAS 12 months	SF-36 12 months
JAW	8	50	4	60	3	80	3	80
EB	9	50	3	70	2	80	2	80
KW	9	60	4	70	3	80	2	80
MM	10	30	3	70	—	—	—	—
SC	8	50	4	80	4	80	2	80
WP	10	60	4	70	—	—	—	—
Mean	9	50	3.6	70	3	80	2.25	80

All patients experienced at least a 50% reduction in pain intensity after the trial period. Consequently, all patients proceeded with the implantation of a non-rechargeable implantable pulse generator (IPG) in the supraclavicular region on the ipsilateral side.

## 4 Discussion

SPG stimulation is a neuromodulation technique that targets the SPG to alleviate pain. The sensory roots formed by the palatine, orbital, and pharyngeal branches form the maxillary branch of the trigeminal nerve. The method we present allows for direct stimulation of the SPG, providing a therapeutic approach for treating TNP localized to the maxillary nerve innervation and palatine areas. The entrance into the pterygopalatine fossa is relatively narrow, allowing for secure fixation of the electrode lead behind the maxillary sinus and anterior to the pterygoid process of the sphenoid, in close proximity to the SPG located within the fossa. For functional neurosurgeons, treatment options for facial pain and headaches linked to trigeminal system pathologies are limited, and SPG stimulation is emerging as a viable alternative. Peripheral nerve stimulation of the supraorbital, infraorbital, or occipital nerves has shown satisfactory results for craniofacial pain (15). An interesting option for treating TNP is the stimulation of the trigeminal ganglion via electrode lead placement in the foramen ovale using a special anchoring system (16). In our opinion, SPG stimulation seems to be more attractive because of the better mode of fixation and immobilization of the lead. Moreover, stimulation of the SPG in most of our patients effectively covered the pain areas of the maxillary (V2) and mandibular (V3) branches, and in one patient (MM), this stimulation was ineffective in the supraorbital (V1) region. In contrast, the other patient (WP) with pain distributed across the V1 and V2 branches experienced successful relief. These findings suggest that afferent stimulation inhibits pain perception primarily in the maxillary nerve (V2) and neighboring mandibular dermatome (V3), and to a lesser extent in the supraorbital (V1) dermatome. For central neuropathic trigeminal pain linked to injury to the second or third neuron of the trigeminal-thalamic pathway, motor cortex stimulation or deep brain stimulation of the periventricular or periaqueductal gray matter, as well as the posterior limb of the capsule or ventral medial thalamic nucleus, may be satisfactory alternatives (6, 7). SPG stimulation offers a less invasive approach than motor cortex stimulation or deep brain stimulation procedures, which carry higher complication risks and are often taxing for patients when performed as two-stage surgeries with trial stimulation periods. The efficacy of these methods for treating TNP, including central pain, is generally variable (6, 7, 18). SPG stimulation has been investigated as a potential treatment for cluster headaches, migraines, post-traumatic headaches, and facial pain disorders (8). In a randomized, sham-controlled study, Schoenen et al. (12) examined SPG stimulation in patients with chronic cluster headaches. The active stimulation group showed significant reductions in attack frequency and pain intensity compared to the sham group. We believe that our approach could be successfully applied to other pain conditions, including cluster headaches and migraines, with a minimally invasive technique and a comparable risk of complications to transoral gingival-implanted miniature stimulators from previous studies (12). Moreover, by adjusting parameters such as stimulation frequency, SPG stimulation may modulate parasympathetic activity in the trigeminal pathway, potentially influencing blood flow in the cranial vessels and neurotransmitter release (17). Tapper explored SPG stimulation as an acute intervention for intractable migraines, showing significant reductions in pain intensity and the need for rescue medications in treated patients (20, 21). SPG stimulation has also been applied to trigeminal autonomic cephalalgia, including refractory cluster headaches, with a significant reduction in headache frequency, intensity, and acute medication use (17). Martelletti et al. (17) and the European Headache Federation have provided an overview of various neuromodulation techniques, including SPG stimulation. The positive effects of SPG stimulation on autonomic headaches are associated with their anatomical connections, as the SPG receives parasympathetic roots from the intermediate nerve via the greater petrosal nerve, which reaches the ganglion through the pterygoid canal. The sympathetic root originates in the superior cervical ganglion, with postganglionic sympathetic fibers passing through the SPG without synapsing alongside the parasympathetic fibers in the vidian nerve (19). SPG stimulation could also potentially benefit conditions such as ischemic stroke or allergic rhinitis (19). Based on our findings, we recommend SPG stimulation using a conventional percutaneous spinal cord stimulation system for TNP refractory to other treatments. Further cohort studies are necessary to evaluate its efficacy in other types of facial pain and headaches.

### 4.1 Limitations

This study was subject to selection bias as SPG stimulation was primarily administered to patients with TNP, which may not fully represent the broader population experiencing persistent facial pain. Additionally, the small sample size and relatively short follow-up period limit the ability to draw definitive conclusions regarding its effectiveness in addressing other facial pain and headache conditions.

## 5 Conclusion

Preliminary results of SPG stimulation in chronic TNP are encouraging. The described method with percutaneous electrode lead inserted in pterygopalatine fossa is minimally invasive and effective with low amplitude of stimulation. The treatment procedure is safe, and the stimulation effect is durable. SPG stimulation can be an attractive alternative to other neuromodulation treatments such as peripheral nerve stimulation (trigeminal g., infraorbital n., supraorbital), motor cortex stimulation, and deep brain stimulation in intractable TNP and other trigeminal autonomic cephalalgias.

## Data Availability

The original contributions presented in the study are included in the article/supplementary material, further inquiries can be directed to the corresponding author.
